# Capturing PM2.5 Emissions from 3D Printing via Nanofiber-based Air Filter

**DOI:** 10.1038/s41598-017-10995-7

**Published:** 2017-09-04

**Authors:** Chengchen Rao, Fu Gu, Peng Zhao, Nusrat Sharmin, Haibing Gu, Jianzhong Fu

**Affiliations:** 1 The State Key Laboratory of Fluid Power and Mechatronic Systems, College of Mechanical Engineering, Zhejiang University, Hangzhou, 310027 China; 2 Key Laboratory of 3D Printing Process and Equipment of Zhejiang Province, College of Mechanical Engineering, Zhejiang University, Hangzhou, 310027 China; 3 Department of Chemical and Environmental Engineering, Nottingham University, Ningbo, 315100 China

## Abstract

This study investigated the feasibility of using polycaprolactone (PCL) nanofiber-based air filters to capture PM2.5 particles emitted from fused deposition modeling (FDM) 3D printing. Generation and aggregation of emitted particles were investigated under different testing environments. The results show that: (1) the PCL nanofiber membranes are capable of capturing particle emissions from 3D printing, (2) relative humidity plays a signification role in aggregation of the captured particles, (3) generation and aggregation of particles from 3D printing can be divided into four stages: the PM2.5 concentration and particles size increase slowly (first stage), small particles are continuously generated and their concentration increases rapidly (second stage), small particles aggregate into more large particles and the growth of concentration slows down (third stage), the PM2.5 concentration and particle aggregation sizes increase rapidly (fourth stage), and (4) the ultrafine particles denoted as “building unit” act as the fundamentals of the aggregated particles. This work has tremendous implications in providing measures for controlling the particle emissions from 3D printing, which would facilitate the extensive application of 3D printing. In addition, this study provides a potential application scenario for nanofiber-based air filters other than laboratory theoretical investigation.

## Introduction

3D printing is gaining increasing popularity due to a fast, cost-effective, and highly customizable manufacturing of products^1^. Fused deposition modeling (FDM) is one of the most commonly used 3D printing methods^1, 2^, which is also known as fused filament fabrication (FFF) or molten polymer deposition (MPD). In this process, a solid thermoplastic filament is melted and extruded from a heated nozzle, and is then deposited onto the growing product^1^. Compared to other 3D printing methods such as stereo lithography (STL), a FDM 3D printer is significantly cheaper^1, 3^, easier to use^2, 3^, and less biologically toxic^1^. Thermoplastics such as, ABS, PLA, and PC, are commonly used in FDM 3D printing due to their low melting temperature^4^. Like any thermal processes of thermoplastics, gas and particles are emitted during FDM 3D printing^3, 5–8^. Kim *et al*. evaluated emissions of ultrafine particles (UFP, particles below 100 nm in diameter) and volatile organic compounds (VOC) from different FDM printers, and reported that the emissions depend on the materials used for filaments^3^. Azimi *et al*. quantified emission rates of UFP and VOC from FDM 3D printing, based on various printers and thermoplastics^5^. Stephens *et al*. measured particle concentrations and emission rates of UFP from the operation of a desktop 3D printer using both acrylonitrile butadiene styrene (ABS) and poly(lactic acid) (PLA)^6^. Zhou *et al*. measured particle concentrations of emissions from ABS 3D printing, and concluded that these particle emissions are highly concentrated in smaller size ranges^7^. Deng *et al*. investigated the impact of processing parameters on particle emissions from 3D printing, and the results showed that the heating process rather than the printing process triggers particle emissions^8^. A brief literature review showed that most particles emitted from 3D printing are of very fine particle sizes^5, 7, 9^, which can be categorized as PM2.5 (particles below 10 μm in diameter). Specifically, ABS has been demonstrated as an easier material to release particle emissions during 3D printing when compared to PLA^3, 5, 6, 8^.

Particulate matter (PM) in air, including UFP, poses a threat to public health^9^ and has been associated with multiple symptoms and diseases^10–12^. In particular, PM2.5 can penetrate lung tissue and migrate to other organs, thus increasing cardiac and respiratory morbidity^13, 14^. In China during 2015, PM2.5 contributed 40.3% to total stroke associated deaths, 33.1% to acute lower respiratory infection (ALRI, <5 yr) associated deaths, 26.8% to ischemic heart disease (IHD) associated deaths, 23.9% to lung cancer (LC) associated deaths, 18.7% to chronic obstructive pulmonary disease (COPD) associated deaths, 30.2% to total deaths combining IHD, stroke, COPD, and LC, and 15.5% to all causes of death, which was estimated by Song *et al*.^15^. As shown in previous studies, 3D printing can be regarded as a source of PM2.5. Multiple pathogenic agents are found within particle emissions from 3D printing^16^. In an indoor environment, the particle concentrations remain high even 20 h after 3D printing^17^. Even for a highly ventilated room, the particle concentrations in the direct vicinity of the 3D printer are still high^18^, potentially jeopardizing the health of the 3D printing operator. Therefore, it is highly desirable to identify an effective method to contain the particle emissions from 3D printing, since there is no published work on this particular topic.

Due to high filtration efficiency, light weight, high transparency, and low air resistance^18, 19^, nanofiber-based air filters are demonstrated to have the potential as one of the promising solutions for the PM2.5 problem^20–22^. A field test in Beijing indicated that the poly acrylonitrile (PAN) transparent air filter would have the best PM2.5 removal efficiency of 98.69% at high transmittance of ~77%. Furthermore, humidity can facilitate PM capture^21^. As an emerging technology, most published research is limited in laboratory scale, since the configuration of air filter has been intensively studied for achieving high filtration efficiency with minimum pressure drop^18–20, 22^. Extending its potential application is highly desirable for a better understanding and further development of this technology. In this study, attention has been focused on using nanofibers for capturing particle emissions from 3D printing, and thereby investigated the generation and aggregation mechanism of the emitted particles. Furthermore, the effects of relative humidity on the aggregation of captured PM were also studied, as relative humidity is one crucial parameter of indoor environment^23^ and controlling humidity might not be easy for any residential building^24^.

## Results

**Figure 1:**
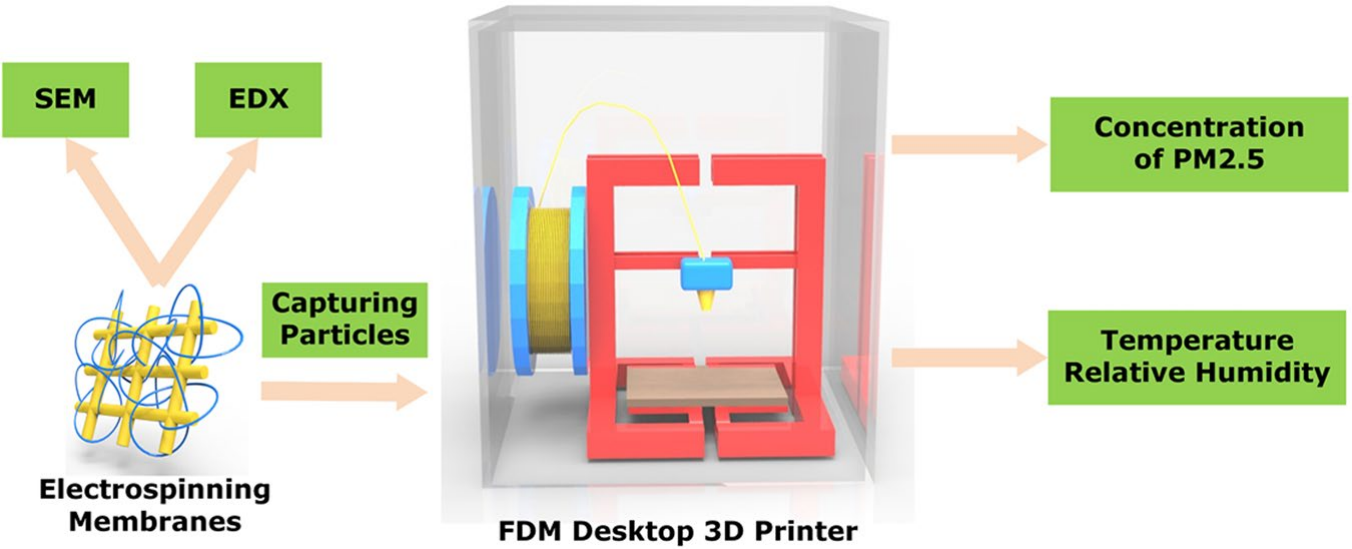
Investigation process of capturing particle emissions from 3D process.

All 3D printing operations were conducted using a FDM desktop 3D printer (model *Up Plus2*, Tier time Co., Ltd., China), which has a printing precision of 0.15 mm. ABS filaments with a diameter of 1.75 mm were used for 3D printing. Since ABS material is one of the most commonly used materials for 3D printing, especially for toxicity assessment^1^. Compared to other materials, more particles are emitted from the ABS 3D printing process^3, 5, 6^. During the 3D printing operations, the platform temperature was retained at 104 °C, and the extrusion temperature was set to 270 °C. The printed product was a 30 × 30 × 30 mm cube (Supplementary Fig.S1), with the following configuration: (1) layer thickness of 0.2 mm, (2) sealing surface angle below 45°, (3) sealing area above 3 mm^2^, and (4) three sealing layers. The 3D printing was conducted in an enclosed testing chamber with a dimension of 0.5 × 0.5 × 0.5 m. The investigation process of capturing particle emissions from 3D process is illustrated in Fig. 1. The chamber was made of acrylic plates, and a hole was drilled on one side of the chamber where an electrospinning PCL nanofiber-based membrane (20 × 20 mm) was covered. The nanofiber-based membranes were sampled at fixed time intervals (1 min), and they were then removed for morphological study. During the sampling time, a small fan was used to evacuate air from the chamber. After removal of one membrane, a new one was replaced. A haze detector (Hanwang Co., Ltd., China) with a PM2.5 detection range of 0–1000 μg m^−3^ and an accuracy below 10% was put aside the 3D printer within the chamber, another identical haze detector was put outside the chamber to monitor the PM2.5 concentrations of the room. The light scattering detector had the advantage of measurement speed, high sensitivity, and reproducibility, which was adopted to test concentrations. Because the process of particle formation was related to the size and quantity of particles, and the light scattering angle can be affected by size and shape of particle^2^, a haze detector was employed to test the concentration. An electronic hygrometer was deployed within the chamber.

The dimension of the room for this experiment was 10 × 5 × 3 m. To exclude any external interferences on the results, all ventilation equipment was closed, and both the door and the windows were sealed using rubber taps. Prior to the experiments, the room was conditioned for 12 h, with the purpose of ensuring effective natural settlement. The chamber was also conditioned for 12 h prior to the experiment, and the PM2.5 concentration in the chamber ranged around 10 μg m^−3^. All experiments were performed in the room and the chamber.

### Particle emissions

No-load test and 3D printing test were studied in this section. The relative humidity (RH) was kept at 70 ± 2%, and the temperature was controlled at 29 ± 2 °C. Both tests were repeated thrice. In the no-load test, the 3D printer was running without ABS filament. The 3D printing operation lasted for 30 min (1,800 s, denoted as “operation”) then, the 3D printer stopped and the experiments were continued for 40 min (2,400 s, denoted as “decay”). The total experiment duration was 70 min (4,200 s). The results of the no-load test shows that PM2.5 concentrations remained within 20 μg m^−3^ in the 70 min experiments (Supplementary Fig. S2). Furthermore, the nanofiber membrane cannot capture particles in this experiment (Supplementary Fig. S3).

**Figure 2:**
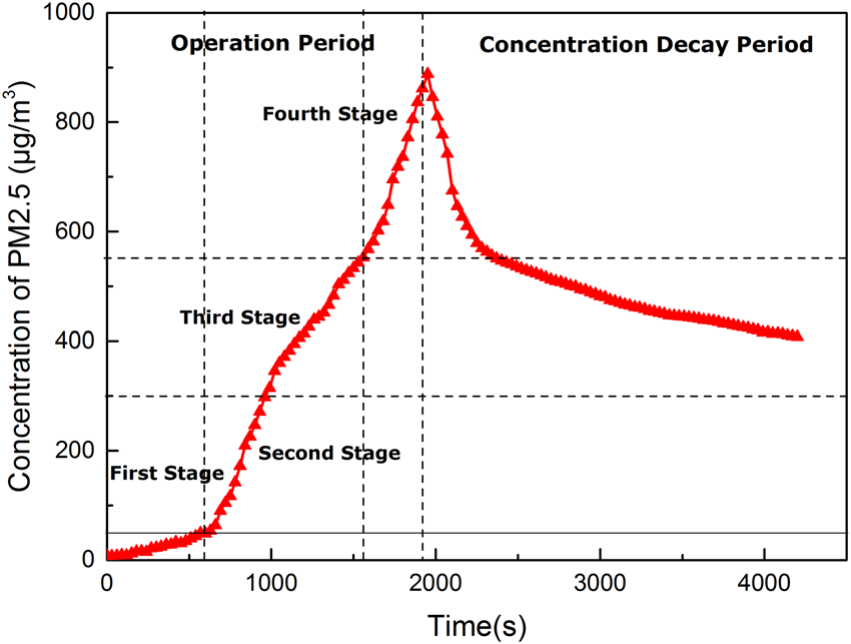
Average PM2.5 concentration of the RH 70% tests.

In the 3D printing test, the average PM2.5 concentration is illustrated in Fig. 2. As shown in Fig. 2, the PM2.5 concentrations remained unchanged during the first two minutes of 3D printing, and increased over time. This means that particle emissions can be attributed to the melting and extrusion of ABS during the 3D printing operation. The peak of PM2.5 concentration was reached at 1,900 s, and the average maximum value of PM2.5 concentration was 900.88 μg m^−3^. After 3D printing stopped, the PM2.5 concentration decreased over time. Based on the results of 3D printing test, the “operation” section can be divided into four distinctive stages: (1) First stage (0–50 μg m^−3^): Before the PM2.5 concentration reached 50 μg m^−3^, the growth rate of the PM2.5 concentration was about 4.3 μg min^−1^ m^−3^. (2) Second stage (50–350 μg m^−3^): The growth rate of PM2.5 concentration was up to 44.76 μg min^−1^ m^−3^, the PM2.5 concentration rapidly increased during this stage. (3) Third stage (350–550 μg m^−3^). The growth rate slowed down as the speed was reduced to 24.67 μg min^−1^ m^−3^. (4) Fourth stage (550–900 μg m^−3^). The growth rate increased to 52.55 μg min^−1^ m^−3^, which shows an index rise.

### Captured particles

**Figure 3:**
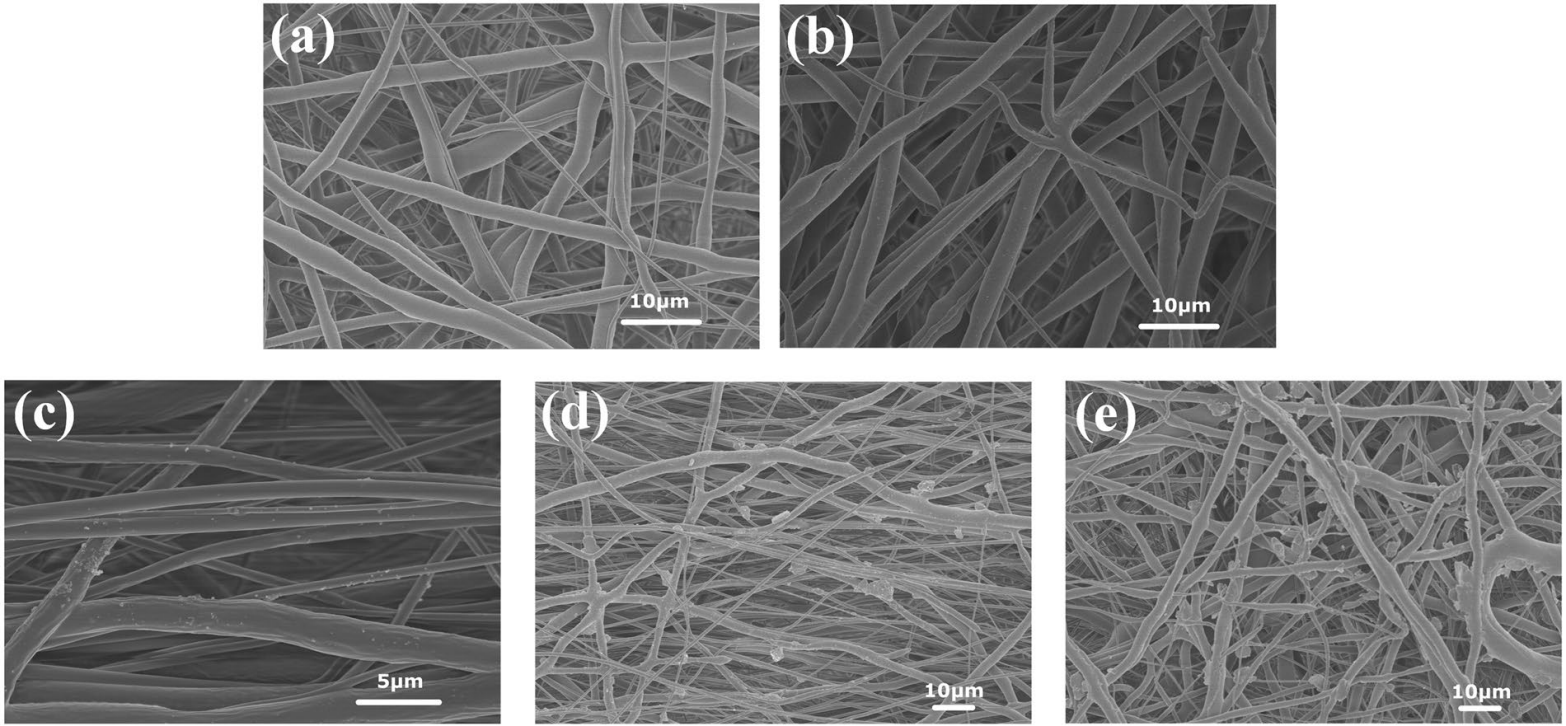
SEM images of emitted particles captured via nanofiber-based membranes in the four different stages: (**a**) original membranes at a magnification of 2 k, (**b**) membranes from the first stage at a magnification of 2 k, (**c**) membranes from the second stage at a magnification of 4.5 k, (**d**) membranes from the third stage at a magnification of 1 k, and (**e**) membranes from the fourth stage at a magnification of 1 k.

The morphological characteristics of the captured particles during 3D printing were investigated. As shown in Fig. 3, with increasing PM2.5 concentration, the size and volume of captured particles in the membranes increased, as different SEM (scanning electron microscope) magnifications were used for the observational study. Figure 3a shows no observable particles on the nanofibers of the membranes prior to sampling. During the first stage, the particles were ultrafine and were not easily found, as shown in Fig. 3b. In the second stage, the submicron particles (650 nm–1 μm) can easily be found on the surface of the nanofibers, as shown in Fig. 3c. During the third stage, only a few the micron scale particles emerged, while the others still remained within the submicron range, as shown in Fig. 3d. During the fourth stage, the volume of micron scale particles significantly increased, as shown in Fig. 3e. The number of particles significantly increased, while the size of the particles also increased significantly.

**Figure 4:**
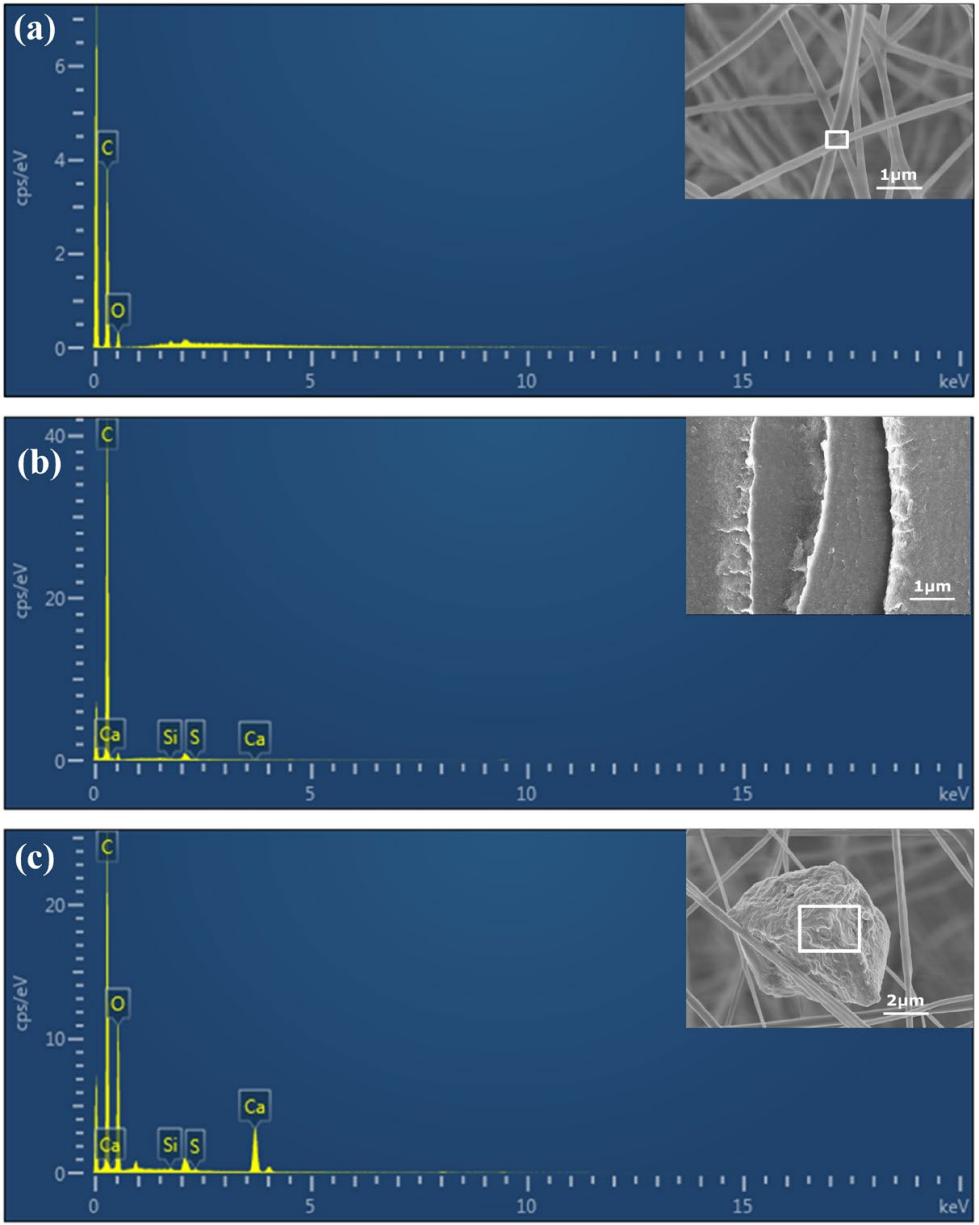
SEM-EDX results: (**a**) of the original nanofibers, (**b**) of ABS filaments, and (**c**) of the captured particles.

At the same time, the results of SEM-EDX analysis for the original nanofibers, ABS filament, and captured particles nanofibers are shown in Fig. 4. The results show that the atomic percentage of different element distribution can be extracted, including carbon elements, oxygen elements, sulfur elements, silicon elements, and calcium elements. The atomic compositions of the original nanofibers are shown in Fig. S4a, and there were only carbon elements and oxygen elements, with atomic percentages of 83.56% and 16.44%, respectively. The atomic compositions of the ABS filament were carbon elements, sulfur elements, silicon elements, and calcium elements, with atomic percentages of 99.87%, 0.08%, 0.03%, and 0.02% (Supplementary Fig. S4b), respectively. The atomic compositions of the captured PM2.5 particles on the nanofibers were five elements, and the atomic percentages of the carbon elements, oxygen elements, sulfur elements, silicon elements, and calcium elements were 61.09%, 35.85%, 0.11%, 0.08%, and 2.88% (Supplementary Fig. S4c), respectively, indicating that the particles could come from the printing material - ABS. The findings indicate that the captured particles are attributed to the melting of the ABS filament during the 3D printing operation. In other words, the nanofiber-based membranes have shown their potential in capturing particle emissions from 3D printing.

### Effects of humidity

3D printing usually takes place in indoor environment. Temperature and humidity are the most common parameters in studying indoor environment, and controlling humidity is more vital in pollution control^23^. Although controlling humidity is highly desirable, it might not always be achievable for any residential building^24^. Considering the massive adoption of 3D printing across the globe, relative humidity was thereby selected as the main controllable parameter in this study. In this section, the temperature was kept at 29 ± 2 °C, and the RH was used as a controllable parameter, controlled at 40%, 50%, 60%, 70%, and 80%. Desiccant and a humidifier using deionized water were used to control the humidity in the chamber, while other testing conditions and measurements remained the same. PCL Membranes sampled from the same time points under different humidity were taken for comparison. The PCL membranes for capturing particles are fabricated in the same equipment, solution, and processing parameters, as shown in Section “Methods”. The diameters of electrospun PCL fibers are not very uniform, and they might vary from nanometers to micrometers^25^.

**Figure 5:**
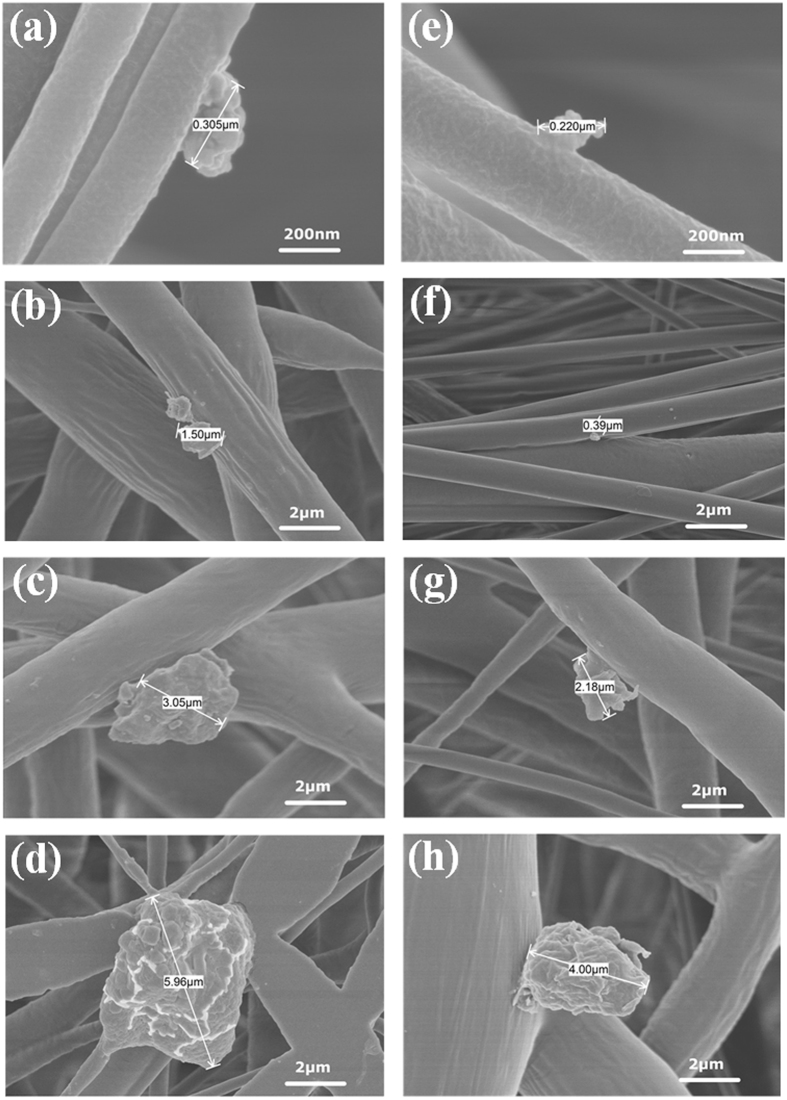
SEM micrographs showing morphological features of the captured particles: (**a**) particles captured during the first stage (RH 70%) at a magnification of 100 k, (**b**) particles captured during the second stage (RH 70%) at a magnification of 10 k, (**c**) particles captured during the third stage (RH 70%) at a magnification of 10 k, (**d**) particles captured during the fourth stage (RH 70%) at a magnification of 10 k, (**e**) particles captured during the first stage (RH 40%) at a magnification of 100 k, (**f**) particles captured during the second stage (RH 40%) at a magnification of 10 k, (**g**) particles captured during the third stage (RH 40%) at a magnification of 10 k, and (**h**) particles captured during the fourth stage (RH 40%) at a magnification of 10 k.

**Figure 6:**
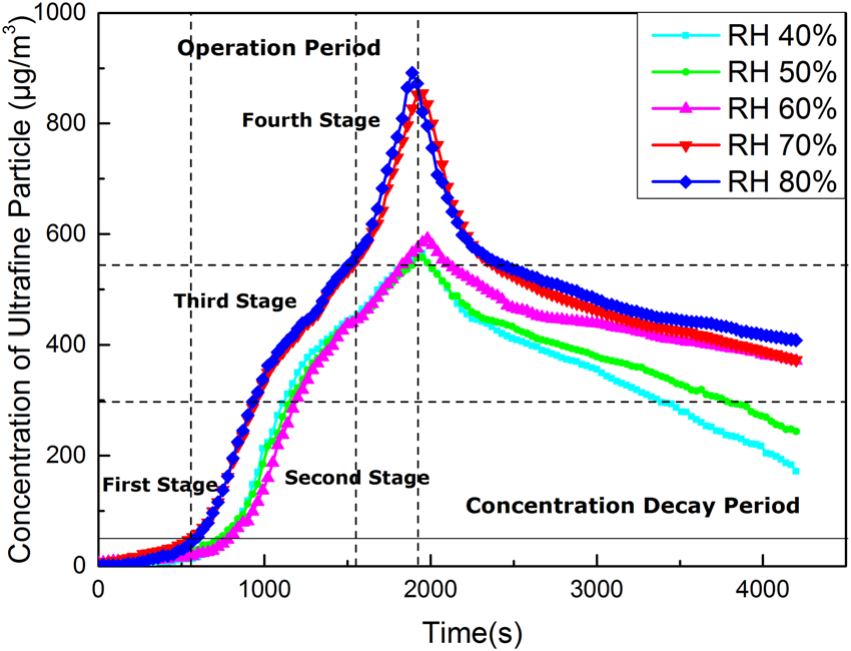
PM2.5 concentrations under different humidity.

The results of the morphological study of RH 70% and RH 40% are presented in Fig. 5 (more evidence is presented in Supplementary Information, see Figs S5 and S6), as different SEM magnifications were used for observing the morphological features of captured particles (the nominal diameter). The PM2.5 concentrations under different humidity are shown in Fig. 6. From these figures, it can be seen that the PM2.5 concentrations and the captured particles in the RH 70% experiment (RH 70% tests) were both significantly higher than those of the RH 40% experiment at all of the four respective stages, implying that higher humidity could facilitate the aggregation of captured particles:

(1) During the first stage, the PM2.5 concentration at RH 70% was about 30.0 μg m^−3^ (see Figs 2 and 6) and the nominal mean diameters of the captured particles were about 300.0 nm, as shown in the Fig. 5a. During the first stage of the RH 40% experiment, the PM2.5 concentration was 25.0 μg m^−3^, which was reduced by 16.7% compared to that of the RH 70% test, and the nominal mean diameters of the particles captured were around 220.0 nm. The difference between the two humidity is not significant at the initial stage, as shown in Fig. 5e.

(2) During the second stage, the PM2.5 concentration at RH 70% was about 240.0 μg m^−3^ (see Figs 2 and 6), which was eight times higher than that of the previous stage. The size and volume of captured particles were significantly larger, as the nominal mean diameters of most particles ranged around 1.5 μm (5 times larger than that of the previous stage (~0.3 μm)), as shown in Fig. 5b. During the second stage of the RH 40% experiment, the PM2.5 concentration was 180.0 μg m^−3^, which was reduced by 25.0% compared to that of the RH 70% test, and the nominal mean diameters of the captured particles were around 0.4 μm, as shown in Fig. 5f. It shows that in the experiments with higher relative humidity, both particle concentration and size increase at a faster rate, which means that the humidity is conducive to the formation of small particles.

(3) During the third stage, the PM2.5 concentration at RH 70% was about 450.0 μg m^−3^ (see Figs 2 and 6), and the aggregated particles had a nominal diameter of 3.0 μm, which was double than that of previous stage, as shown in Fig. 5c. During the third stage of the RH 40% experiment, the PM2.5 concentration was 400.0 μg m^−3^, which was reduced by 11.1% compared to that of the RH 70% test, and the nominal mean diameters of the particles captured were around 2.2 μm, as shown in Fig. 5g. At this stage, the particle concentration and size maintain a similar growth rate under different humidity.

(4) During the fourth stage, a similar pattern was again observed, as the number of larger aggregated particles significantly increased. The PM2.5 concentration at RH 70% was about 720.0 μg m^−3^ during the fourth stage (see Figs 2 and 6), and the nominal mean diameter of the captured particles reached about 6.0 μm, as shown in Fig. 5d. During the fourth stage of the RH 40% experiment, the PM2.5 concentration was 500.0 μg m^−3^, which was reduced by 30.5% compared to that of the RH 70% test, and the nominal mean diameters of the particles captured were around 4.0 μm, as shown in Fig. 5h. At this stage, the particle concentrations of different humidity conditions are significantly different, as well as their sizes, that indicates the humidity imparts a great impact on the concentration and the size of particle emissions.

In order to prove that the humidity on the particle concentration does have an impact, this section sets the humidity as the only variable, and the evolution of particle concentration during 3D printing is studied. As shown in Fig. 6, the five curves show the change in particle concentration under five different humidity levels. The trends of the PM2.5 concentrations under the different relative humidity values were generally identical in the four stages. None of the curves shows any obvious differences at RH 40%, RH 50%, and RH 60%. When the relative humidity reached 70%, the PM2.5 concentrations were significantly higher than those at lower humidity, while little increments in PM2.5 concentrations could be observed when the relative humidity further increased to 80%.

## Discussion

**Figure 7:**
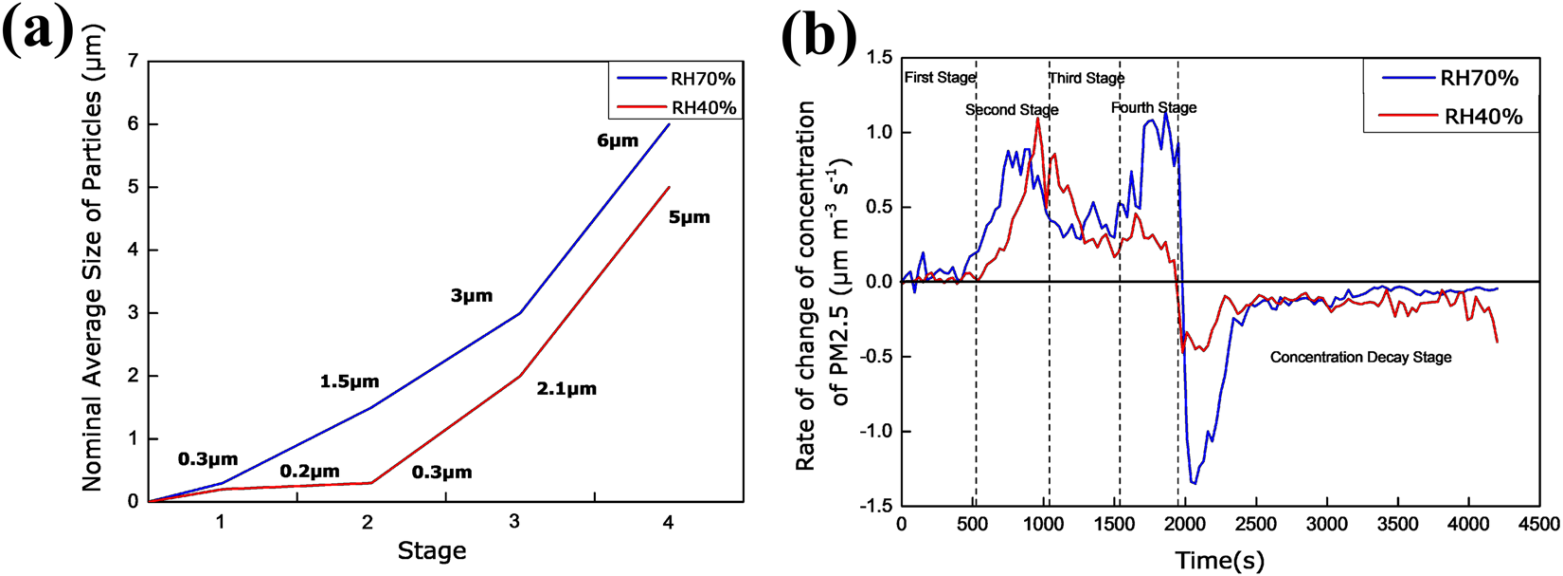
Capture particle and concentration data analysis: (**a**) Nominal average size of particles in different stage, and (**b**) Changing rates of the PM2.5 concentration under different RH.

In this section, the generation and aggregation mechanism of the particles emitted from 3D printing is discussed. Generally, there are three routes of submicron particles formation including homogeneous nucleation, heterogeneous nucleation and collision- coalescence^26^. The changes in the nominal average sizes and concentration changing rates of the captured particles are summarized in Fig. 7. The Fig. 7a shows the nominal average size of particles over different stages, and Fig. 7b shows the changing rates of the PM2.5 concentration under different humidity. From the images, the increasing particle size over time and the fluctuating particle concentration can be clearly observed. In the first stage, the PM2.5 concentrations were low and the sizes of the captured particles were small, since the particles were just generated and arrived on the nanofibers. These small particles are denoted by “building units”. In the second stage, a higher particle concentration was achieved at higher relative humidity. At RH 40%, the size of particles did not increase obviously, while the PM2.5 concentration changing rates increased. This resulted in an increase in the number and spreading of building units, as more building units were aggregated on the nanofibers. In the third stage, the PM2.5 concentrations increased, but the increasing trend had slowed down. Due to Brownian random motion, the particle collision was intensified, which led to a reduction in the number of particles and an increase in the diameter of the captured particles. The particle coagulation and diffusion took place during the stage. When the larger particles coagulated and settled on the nanofibers, a significant reduction in the PM2.5 concentrations was observed. The phenomena was compatible to the findings of Kim *et al*.^3^. In the fourth stage, the concentration and sizes of particles were increasing continuously and simultaneously, due to the critical supersaturation was reached and the imbalance of kollisionspartner of small particles was strengthened by Brownian motion. The results were comparable with the findings of previous literature^27, 28^. After 3D printing stopped, the PM2.5 concentration reading still rose within a few seconds, which could attribute to the suspended particles had not spread to the detection position.

**Figure 8:**
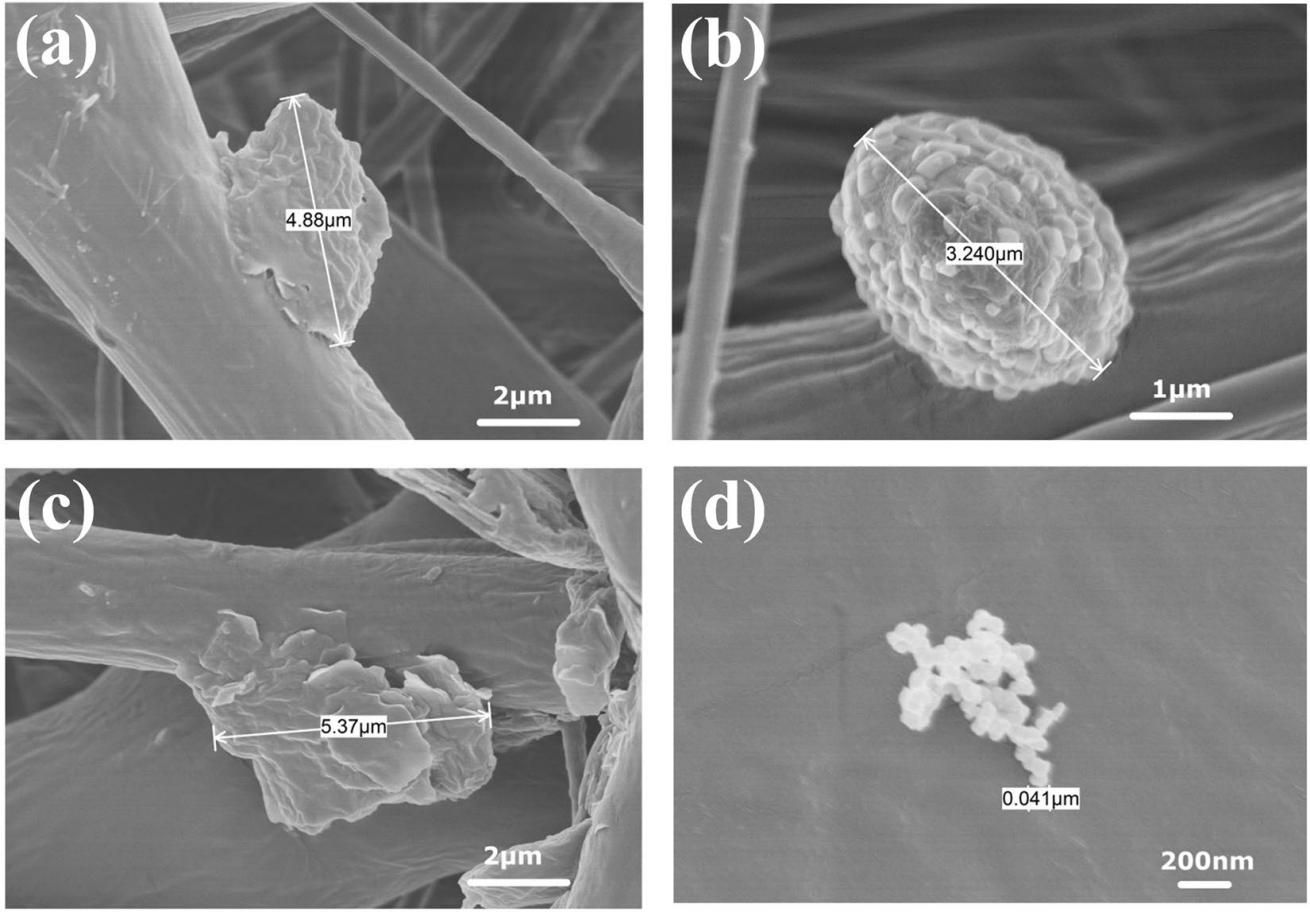
SEM micrographs of different morphologies of particles at the fourth stage: (**a**) morphology of heterogeneous nucleation at a magnification of 10 k, (**b**) morphology of homogeneous nucleation with building units at a magnification of 20 k, (**c**) morphology of sheet building units at a magnification of 10 k, and (**d**) morphology of spherical building units at a magnification of 50 k.

The mechanism of particle aggregation were included the SEM morphological study as well. According to the previous study^29, 30^, the number of spherical particles formed via homogeneous nucleation was much lower than the sheet particles formed via heterogeneous nucleation, which indicated that the energy barrier of homogeneous nucleation was significantly higher than that of heterogeneous nucleation. Therefore, the occurrence of homogeneous nucleation is much more difficult in the same over-saturation conditions. In the fourth stage, during the nucleation process, typical micron-sized particles were shown in the Fig. 8. Heterogeneous nucleation (see Fig. 8a) and homogeneous nucleation (see Fig. 8b) were found to be coexist, and the sheet and spherical building units were also coexisted, as shown in Figs 8c and d. During the 3D printing process, with increasing building units, the submicron particles constantly generated through collide-coagulation and self-growth, and thereby led to an increased size and quantity of the aggregated particles. The finding is comparable to the work of Zhu *et al*.^26^ on particle emissions from heavy-duty diesel traffic.

Based on the PM2.5 concentration curves under different humidity (see Fig. 6), it can be concluded that relative humidity can influence the PM2.5 concentration to a certain extent. With increasing relative humidity, the PM2.5 concentrations increased accordingly, and the generation of small particles was at an accelerated speed, as the similar effect of humidity was also observed by Deng *et al*.^8^. A comparison of the particle sizes of different humidity and stages (see Fig. 5) showed that the growth of micron-sized particles was relatively slow under low humidity. Possible reasons could be: the particles had a low content of water vapor during the formation process, therefore the embryos could not be formed during the coagulation process, inducing a smaller volume of micron-sized particles under low humidity^31^. In addition, increasing relative humidity facilitates the consolidation of the gaseous particles. Therefore, increasing relative humidity and concentration would increase the diameter of captured particles through intensifying the collisions between particles which facilitate the merging of small particles and the formation of large particles^32, 33^.

The contribution of this study is threefold. First, the pattern of particle emissions from FDM 3D printing has been further investigated, as different stages of PM2.5 generation and the effects of humidity are identified. Second, the feasibility of using PCL based nanofiber membranes to capture PM2.5 from 3D printing has been proved. Third, the morphological characteristics of captured particles has been studied, as well as the aggregation of particles over time and the effects of relative humidity. The study provides tremendous implications to a board audience, and provides a plausible measure to counter one of the major weakness of the 3D printing process - particulate emission. With the extensive adoption of 3D printing, the nanofiber-based membranes would certainly draw an increasing attention for the course of occupational health and safety. This study offers an application scenario for nano-filters, and it extends the scope of the research on this particular field, since the extent literature focuses only on laboratory investigations^18–20, 22^.

## Methods

### Raw materials

ABS was purchased from Chimei Co., Ltd., China with a trademark of PA-757. The material was extruded via single screw extruder with a die of 1.75 mm at a temperature profile of 200 °C–210 °C–220 °C –200 °C, and the filament was used for 3D printing. PCL (*Mw* = 90,000) was purchased from Sigma-Aldrich Co., Ltd., UK. N,N-dimethylformamide (DMF) and dichloromethane (DCM) were supplied by the Shanghai Chemical Reagents Co., Ltd., China. All chemicals were of reagent grade and were used as received without any further purification or modification.

### Fabrication of membranes

The nanofiber was prepared via electrospinning, which is a common method used for the fabricating of nanofibers for air filters^18–22^. 12 wt.% of PCL solution was prepared using DMF and DCM with a stirring speed of 200 rpm for 24 h, and the mass ratio of DMF and DCM in the solutions was kept 1: 4. The solution was evacuated using a 10 mL plastic syringe, which connected to a metal capillary (*d* = 0.5 mm) via a plastic tube. The membranes of nanofibers were fabricated using TL-01 electrospinning equipment (Tongli Co., Ltd, China) at an ambient temperature of 35 °C and a relative humidity of 50%. The processing parameters were set as follows: (1) an injection rate of 3.5 ml h^−1^, (2) an applied voltage of 10 kV, (3) a tip-to-collector distance of 15 cm, and (4) the nozzle at a linear speed of 2.3 mm min^−1^. Then, the membranes were dried in a vacuum drying oven at 30 °C for 8 h to vapor all solvent, and 99.9% nitrogen gas was purged into the oven as protective gas. An SEM image of the prepared membrane is shown in Supplementary Fig. S7, showing that the diameter of the fibers were below 500 nm, which is in accordance with the definition of a nanofiber^22^.

### Morphological study

The morphological study on the nanofiber-based membranes (before and after the particle capturing) was carried out via a Hitachi UHR FE SU8000 SEM at an acceleration voltage of 3 kV. Moreover, a SEM-EDX (Energy Dispersive X-Ray Spectroscopy) was also performed. The scanned zones were randomly taken from the membranes, averaging eight sampling zones per membrane. To prevent a drift phenomenon of the samples in SEM, the samples were thoroughly dried in the oven for 1 h, to enable clear and stable observation of the morphology of the original particles.

## Electronic supplementary material

Supplementary Information for Capturing PM2.5 Emissions from 3D Printing via Nanofiber-based Air Filter
