# Supplementary material for: Capturing PM2.5 Emissions from 3D Printing via Nanofiber-based Air Filter
Source: Sci Rep. 2017 Sep 4;7:10366. doi: 10.1038/s41598-017-10995-7 (PMC5583319; doi:10.1038/s41598-017-10995-7)
Supplement: Supplementary file 1 — Supplementary Information for Capturing PM2.5 Emissions from 3D Printing via Nanofiber-based Air Filter [file 41598_2017_10995_MOESM1_ESM.pdf]

Supplementary Information for Capturing PM2.5 Emissions from 3D Printing via Nanofiber-based Air Filter

Chengchen Rao[1,2], Fu Gu[3], Peng Zhao[1,2,*], Nusrat Sharmin[3], Haibing Gu[1,2], Jianzhong Fu[1,2]

(*1. The State Key Laboratory of Fluid Power and Mechatronic Systems, College of Mechanical Engineering, Zhejiang University, Hangzhou 310027, China.*

2. Key Laboratory of 3D Printing Process and Equipment of Zhejiang Province, College of Mechanical Engineering, Zhejiang University, Hangzhou 310027, China.

3. Department of Chemical and Environmental Engineering, Nottingham University, Ningbo 315100, China.

**Corresponding to: Peng Zhao, e-mail: pengzhao@zju.edu.cn*)

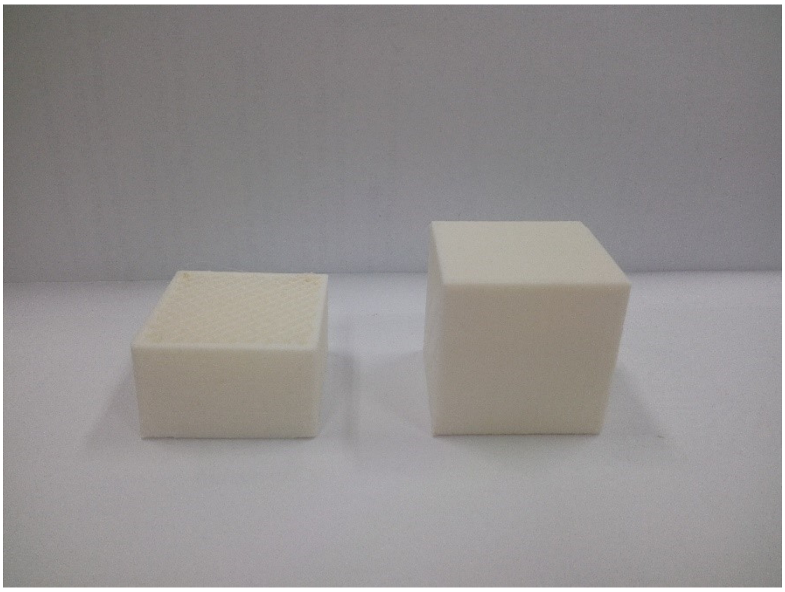

Fig.S1. Examples of 3D printed samples: The left is a sample which was printed during 30 min, and the right shows a 30 × 30 × 30 mm cube.

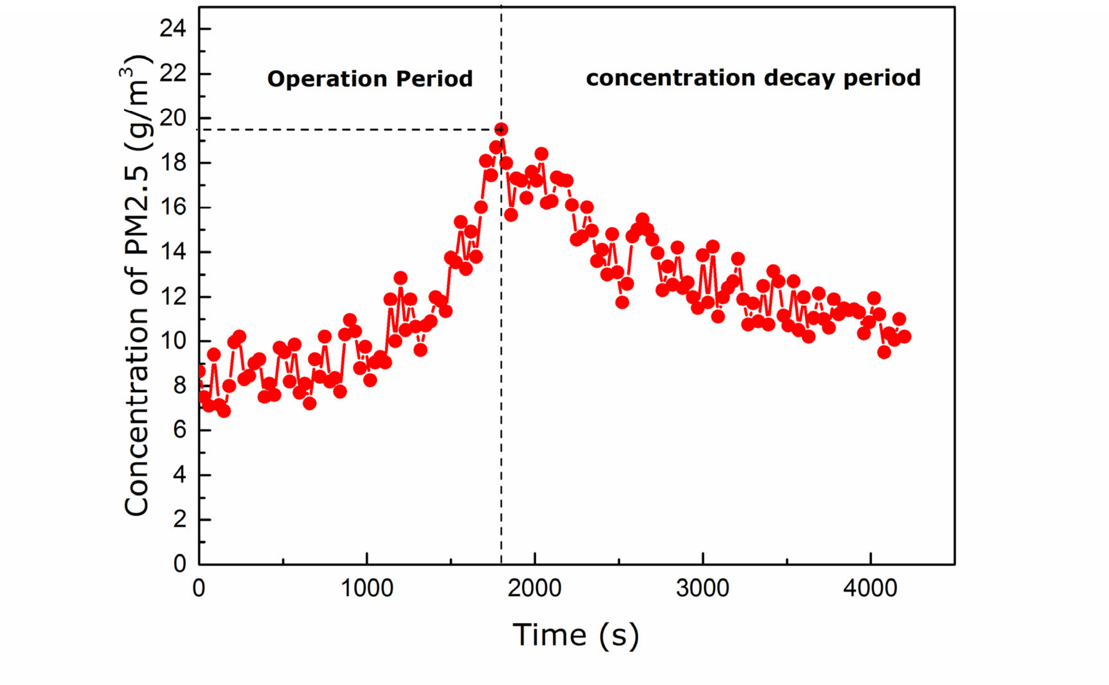

Fig.S2. Average PM2.5 concentrations of the no-load test.

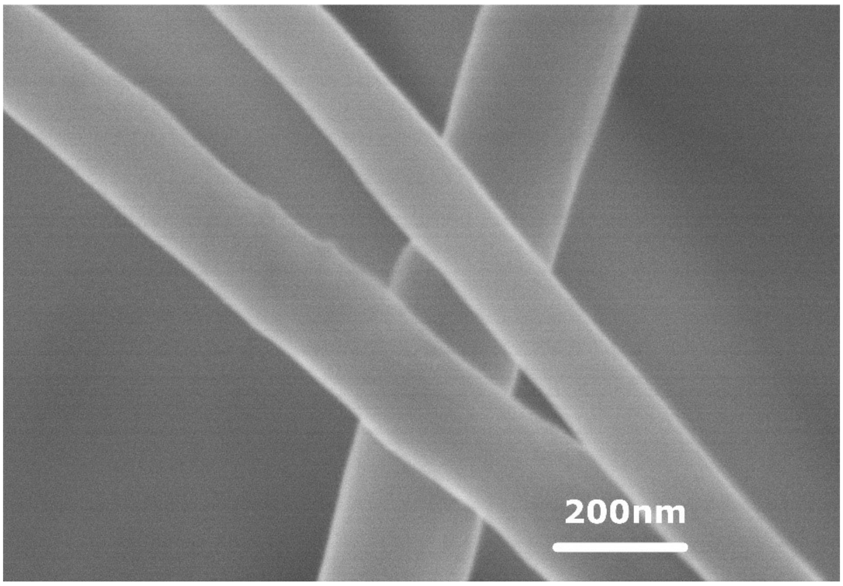

Fig.S3. Captured particle SEM image of nanofiber membrane in no-load test.

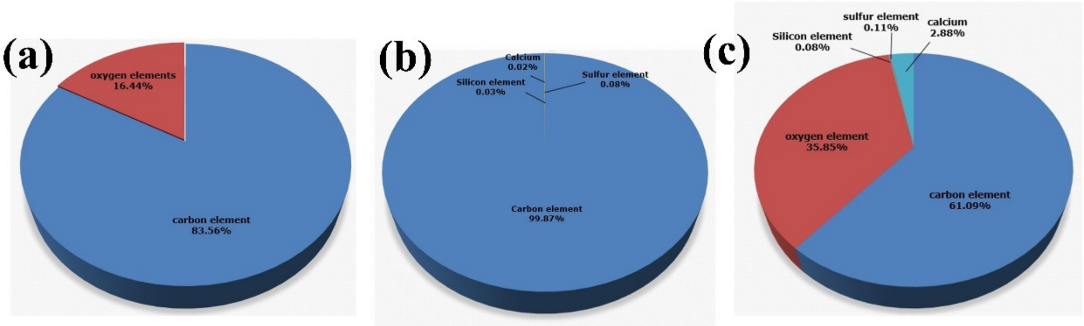

Fig.S4. The atomic compositions obtained via SEM-EDX scans: (**a**) atomic composition of original nanofibers, (**b**) atomic composition of ABS filament, and (**c**) atomic composition of captured particles on the nanofibers.

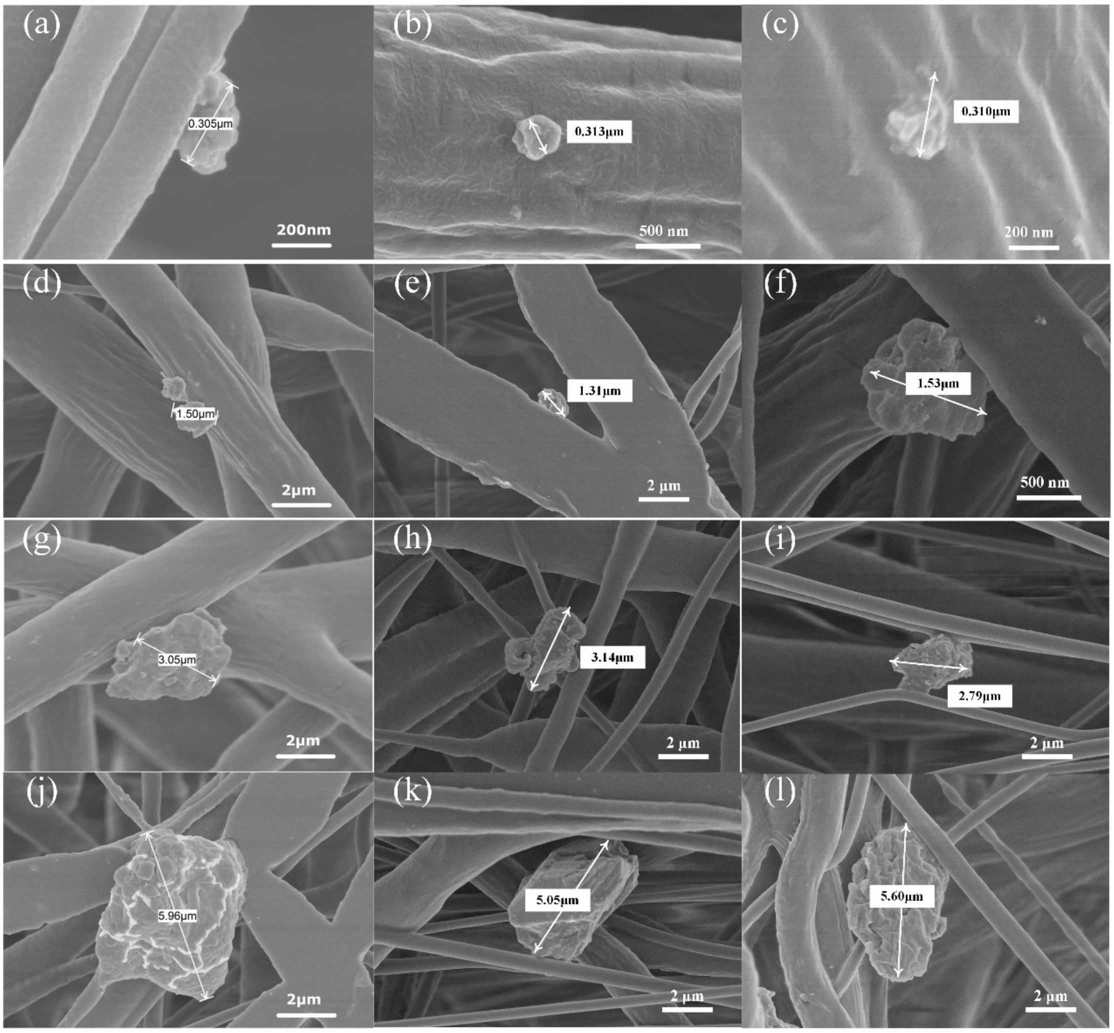

Fig.S5. SEM micrographs showing morphological features of the captured particles: (**a**)-(**c**) particles captured during the first stage (RH 70%), (**d**)-(**f**) particles captured during the second stage (RH 70%), (**g**)-(**i**) particles captured during the third stage (RH 70%), (**j**)-(**l**) particles captured during the fourth stage (RH 70%).

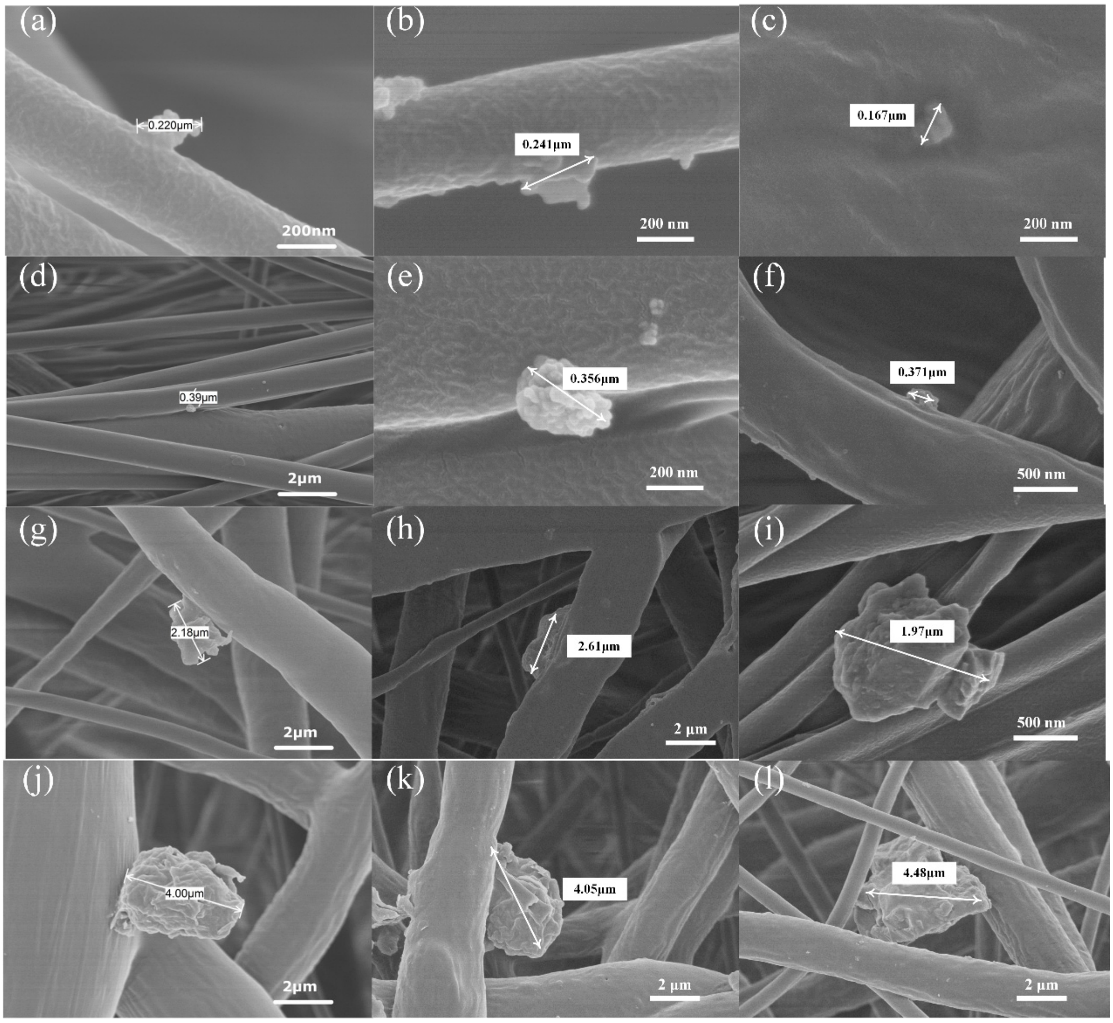

Fig.S6. SEM micrographs showing morphological features of the captured particles: (**a**)-(**c**) particles captured during the first stage (RH 40%), (**d**)-(**f**) particles captured during the second stage (RH 40%), (**g**)-(**i**) particles captured during the third stage (RH 40%), (**j**)-(**l**) particles captured during the fourth stage (RH40%).

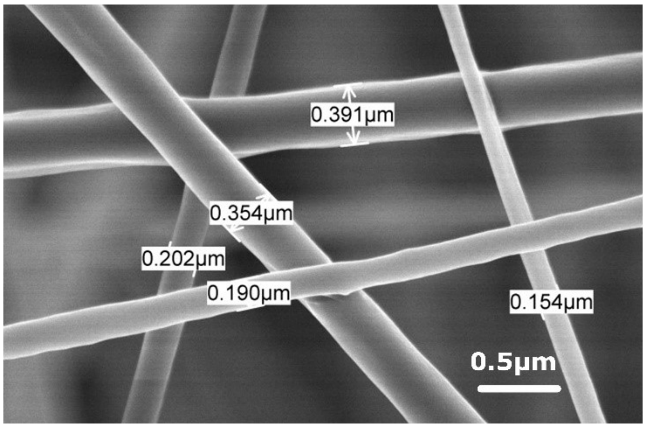

Fig.S7. SEM image of a RH 70% electrospinning membrane at a magnification of 30 k.
